# Rapid eye movement dependency is associated with increased inflammatory activity in obstructive sleep apnea syndrome

**DOI:** 10.1002/brb3.3546

**Published:** 2024-06-06

**Authors:** Asli Akyol Gurses, Utku Ogan Akyildiz

**Affiliations:** ^1^ Division of Clinical Neurophysiology, Department of Neurology, Faculty of Medicine Gazi University Ankara Turkey; ^2^ Department of Neurology, Faculty of Medicine Aydin Adnan Menderes University Aydin Turkey

**Keywords:** inflammation, platelet distribution width, REM‐dependent OSAS, REM‐related OSAS, systemic immune‐inflammation index

## Abstract

**Objective:**

Rapid eye movement (REM)‐dependent obstructive sleep apnea syndrome (OSAS) is a specific subtype of OSAS having some phenotypic characteristics like a preference for a younger age, female gender, and milder severity. Such favorable features could make it possible to consider an overall benign course for this phenotype. However, accumulating data introduced its association with several cardiometabolic and vascular disorders recently. The primary objective of this study was to address the disease from the inflammation perspective and evaluate the potential inflammatory status in this variant via two accessible blood parameters: platelet distribution width (PDW) and systemic immune‐inflammation index (SII). The secondary aim was to investigate whether this status, together with other disease characteristics, demonstrates consistency under different definitions of REM‐dependent OSAS published previously.

**Patients and methods:**

The medical records of 35 patients with mild‐to‐moderate REM‐dependent OSAS, 35 age‐ and sex‐matched patients with REM‐independent OSAS, and 25 non‐OSA controls were retrospectively analyzed. Baseline features, polysomnographic characteristics, PDW, and SII were compared between the groups. Secondly, the analyses were repeated using different definitions of REM‐dependent OSAS. Bivariate analyses were performed, and a multiple stepwise regression model was applied to adjust for body mass index (BMI) and cardiovascular risk (CVR) factors.

**Results:**

Mean PDW and SII were increased in patients with REM‐dependent OSAS as compared to non‐OSA controls (*p* = .022 and .029). The significance remained stable after adjustment for BMI and CVRs and was consistent according to different definitions. The Comparison of patients with REM‐independent OSAS and non‐OSA controls, as well as the two different subtypes of OSAS, did not yield significance.

**Conclusion:**

Based on the current findings, patients with REM‐dependent OSAS appear to be susceptible to inflammation and should be carefully monitored for the negative consequences of that issue. To our knowledge, this study is the first to evaluate SII and PDW in REM‐dependent OSAS.

## Graphical abstract

REM‐dependent OSAS has some typical characteristics that distinguish it from stage‐independent OSAS. These include a preference for younger age, female gender, and milder disease severity. Although these features suggest a benign course for this phenotype, recent literature shows that it is also associated with various cardiometabolic consequences. In our retrospective study, we found that PDW and SII, two accessible blood parameters, indicate an increased inflammatory status in this subtype. Our results highlight the need for close follow‐up in patients with REM‐dependent OSAS, as increased systemic inflammation can have negative consequences.

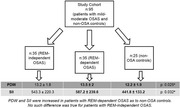

## 1 INTRODUCTION

Obstructive sleep apnea syndrome (OSAS) is a common disorder characterized by partial or total restrictions of the upper airway during sleep that result in desaturation and/or arousal and pave the way for adverse cardiovascular outcomes in the long term. Male gender, advanced age, and excess weight are the among well‐defined risk factors. Rapid eye movement (REM)‐dependent OSAS is a separate subtype in which the abovementioned respiratory events predominantly or entirely occur during the REM stage. It accounts for approximately 12%–36% of all cases and is distinguished by the preference of younger age, female gender, and milder disease level (Haba‐Rubio et al., 2005; Mano et al., 2019; Mokhlesi, 2012; Nieto et al., 2000).

Clinical implications of REM‐dependent OSAS have accumulated in recent years despite ongoing controversy in terms of definition and the need for therapy (Rishi & Rishi, 2021). Theoretical assumptions of ischemic preconditioning due to longer apneas and profound desaturation considered typical for this variant do not seem to serve as a protective mechanism as proposed before (Garvey et al., 2009; Ryan et al., 2005).

The demonstration of an independent relationship between REM‐dependent OSAS and prevalent hypertension in Wisconsin and MAILES cohorts, as well as poor glycemic control in another study population, raised the question of whether these subset of OSAS patients are suffering from an active inflammatory process and thus more prone to atherosclerotic and cardiometabolic consequences (Appleton et al., 2016; Grimaldi et al., 2014; Mokhlesi et al., 2014). Recently, Sangchan et al. (2023) reported an association between REM‐OSA and the tendency to develop common cardiometabolic diseases, hypertension in particular, and Koo et al. (2020) defined a cutoff value of >15/h for apnea–hypopnea index (AHI) during REM as an independent predictor of metabolic syndrome.

In this retrospective study, we investigated the inflammatory status of patients who were examined for the suspicion of OSAS. Our main aim was to determine if there were any differences in REM‐dependent OSAS, REM‐independent OSAS, and non‐OSA controls. To achieve this, we evaluated two well‐defined markers: platelet distribution width (PDW) and systemic immune‐inflammation index (SII), which provide valuable information from platelet and leukocyte series. Since milder patients form a considerable proportion of REM‐dependent OSAS, our analyses were conducted on patients with mild‐to‐moderate disease levels. The secondary aim was to investigate the consistency of clinical and laboratory findings according to different definitions of REM‐dependent OSAS. For this second purpose, we reclassified and reanalyzed our study population in accordance with two other criteria, referred to as “traditional” and “strict” definitions, which were published previously (Conwell et al., 2012).

## 2 MATERIALS AND METHODS

### 2.1 Patients and definitions of REM‐dependent OSAS

The medical records of patients who underwent all‐night diagnostic polysomnography in the sleep laboratory of a single center between June 2017 and February 2019 were reviewed retrospectively. The patients were first evaluated by a sleep specialist (Dr. Asli Akyol Gurses) in the outpatient clinic of the Clinical Neurophysiology and Sleep Disorders Department and then referred to the sleep laboratory for diagnostic polysomnography in case of complaints suggestive of OSAS (snoring, witnessed apnea, excessive daytime sleepiness, etc.), Although some patients had no clinical complaints, those with a body mass index (BMI) greater than 30 kg/m2 were ordered to undergo a diagnostic study. This was necessary for those who had requested a driver's license on admission since obtaining or renewing a driver's license in our country requires documented exclusion of obstructive sleep apnea syndrome (OSAS) for individuals with a BMI greater than 30 kg/m2. Following referral to the sleep laboratory during this first visit, which allows the sleep technician to obtain contact information, introduce the laboratory conditions to the patients, and explain the details of the procedure, the diagnostic study was generally performed within 2–4 weeks. After diagnostic polysomnography, patients were either diagnosed with OSAS (AHI ≥ 5/h) according to International Classification of Sleep Disorders‐3 (ICSD‐3) criteria or accounted as non‐OSA controls (AHI < 5/h) (American Academy of Sleep Medicine, 2014; American Academy of Sleep Medicine Task Force, 1999). Disease severity was graded depending on the AHI.

Age, gender, body‐mass index (BMI), scores of Eppworth Sleepiness Scale (ESS), history of smoking, comorbid diseases, cardiovascular risk factors including hypertension, diabetes mellitus, and hyperlipidemia; polysomnographic features including respiratory indices (AHI_Total_, AHI_REM_, AHI_NREM_, oxygen desaturation index [ODI], desaturation duration [spO2 < 90%], minimum spO2, and average spO2) and sleep parameters (total recording time [TRT]; total sleep time [TST]; sleep efficiency [SE]; sleep onset latency [SOL]; arousal index [AI]; wake after sleep onset [WASO]; proportion of N1, N2, N3, and REM stages; and REM and N3 stage duration); and inflammatory markers derived or calculated from hemogram studies including PDW and SII were recorded.

Two sets of exclusion criteria were determined during the current study: one was clinical and the other was polysomnographic. Clinical exclusion criteria were as follows: diagnosis of any hematological and autoimmune systemic disorders/chronic inflammatory conditions; overt cardiovascular and cerebrovascular diseases (acute myocardial infarction [MI], arrhythmias and atrial fibrillation [AF], congestive heart failure [HF], and stroke) (Benjamin et al., 2018); obesity hypoventilation syndrome; active infectious condition at the time of blood sampling; and medication that can influence hemogram parameters. Exclusion criteria based on the polysomnographic assessment included a severe level of OSAS (AHI ≥ 30/h) (American Academy of Sleep Medicine Task Force, 1999) and an unsatisfactory diagnostic study (sleep efficiency < 50%). Note that 30 minutes of REM amount in the presence of “AHIREM/AHINREM ≥ 2” was considered a prerequisite for the diagnosis of REM‐dependent OSAS in this study; therefore, patients with an inadequate (<30 minutes) REM duration were also excluded during the identification of REM‐dependent OSAS group (Mokhlesi & Punjabi, 2012). After the selection of eligible patients for REM‐dependent OSAS(primary) (see below for a further explanation of “primary definition”), the same number of age–sex‐matched patients with REM‐independent (AHIREM/AHINREM < 2) OSAS and a similar number of age–sex‐matched non‐OSA controls (AHI < 5) satisfying the inclusion criteria were consecutively recruited. Three separate definitions used for REM‐dependent OSAS during this study were as follows: (1) “overall AHI ≥ 5 and AHI_REM_/AHI_NREM_ ≥ 2” which referred to as “primary definition” (a tightened version of the first suggestion with adequate −30 min‐REM period requirement) (Haba‐Rubio et al., 2005; Mokhlesi & Punjabi, 2012); (2) “overall AHI ≥ 5, AHI_REM_/AHI_NREM_ ≥ 2, and AHI_NREM_ < 15” which referred to as “traditional definition”; and (3) “overall AHI ≥ 5, AHI_REM_/AHI_NREM_ ≥ 2, and AHI_NREM_ < 8” which referred to as “strict” definition (Conwell et al., 2012; Haba‐Rubio et al., 2005). The “strict” definition introduced by Conwell et al. (2012) also included “minimum 10.5 min of REM sleep duration” as an additional criteria in the original form, but our duration limit for REM sleep was at least 30 minutes and kept constant for all three definitions during this study.

To test the primary hypothesis, we used the primary criteria in the first step. For exploring the second objective, we applied two other definitions to the same cohort. The local ethics committee approved this retrospective study.

### 2.2 Blood analysis

Blood samples for hemogram analysis were taken from every patient during the first visit to the outpatient clinic in the morning following overnight fasting. The samples were collected into EDTA tubes and automatically analyzed via SYSMEX XE‐2100 automated hematology analyzer (Sysmex Corporation). PDW values were derived from the hemogram recorded, and SII values were calculated by the following formula: “platelet count × neutrophil count/lymphocyte count (×10^9^/L)” (Hu et al., 2014).

### 2.3 Polysomnography

All patients underwent full‐night polysomnography after being informed about the details and importance of the procedure and potential risks during the monitorization. Patient safety was prioritized throughout the entire process, from entry to the laboratory to discharge. At least 6 h of monitoring was provided for each patient after obtaining informed consent.

The polysomnographic records included electrooculogram (EOG), six‐channel electroencephalogram (EEG), chin and leg electromyogram (EMG), electrocardiogram (ECG), thoracic and abdominal respiratory effort, oronasal thermal and nasal airflow, pulse oximetry, and body position. For the EEG montage, the recommended lead connection was based on American Academy of Sleep Medicine (2014) v2.4, with main electrodes placed at F4, C4, O2, and M1 positions to display F4‐M1, C4‐M1, and O2‐M1 channels. Backup electrodes were also placed at F3, C3, O1, and M2 positions to display F3‐M2, C3‐M2, and O1‐M2 channels, respectively (Berry et al., 2017). The Nox medical program was used to collect data, which were then manually scored and interpreted by the same sleep specialist and clinical neurophysiologist (Dr. Asli Akyol Gurses) according to American Academy of Sleep Medicine (2014) v2.4 guidelines (Berry et al., 2017). In regard to respiratory events, an apnea was identified as a ≥90% reduction in the baseline amplitude of the oronasal thermistor airflow signal for a minimum of 10 seconds. On the other hand, hypopnea was identified as a ≥30% reduction in the amplitude of the nasal airflow signal lasting at least 10 seconds and resulting in ≥3% oxygen desaturation or arousal.

Patients with an AHI of ≥5 and <15 were classified as having mild OSAS, while those with values between ≥15 and <30 were classified as having moderate OSAS (American Academy of Sleep Medicine, 2014; American Academy of Sleep Medicine Task Force, 1999). Non‐OSA controls were individuals with similar complaints or no complaints but referred for screening due to obesity and found to have an AHI of <5 after a diagnostic study. Among OSAS patients (AHITotal ≥ 5/h), a diagnosis of REM‐dependent OSAS was made according to three different definitions, as mentioned above.

### 2.4 Statistical analysis

Statistical analysis was conducted using the IBM SPSS.20 (SPSS, Inc.) package program. The normality of the data was tested using the Shapiro‐Wilk test. Continuous parameters were presented as mean ± SD or median (minimum–maximum) depending on their distribution. Categorical variables were given as percentages and evaluated by the Chi‐squared test. Comparisons between two groups for continuous parameters were performed via independent samples t‐test or Mann–Whitney U‐test according to the normality of the data. When the groups were more than two, an analysis of variance or Kruskal–Wallis test was applied according to the distribution. In the case of overall significance, a post hoc Bonferroni test was applied for pairwise comparisons where appropriate. Simple correlations between blood parameters (PDW and SII) and polysomnographic indices (AHI and ODI) were evaluated using Pearson correlation test.

To explore the independent associations of inflammatory markers (PDW and SII), a multiple stepwise regression model was applied, which included BMI, cardiovascular risk (which referred to the presence of any of the following: hypertension, diabetes mellitus, hyperlipidemia, and smoking history), and REM dependency (which referred to “AHI_REM_/AHI_NREM_ ≥ 2”). A two‐tailed p‐value of <.05 was considered statistically significant.

## 3 RESULTS

**FIGURE 1:**
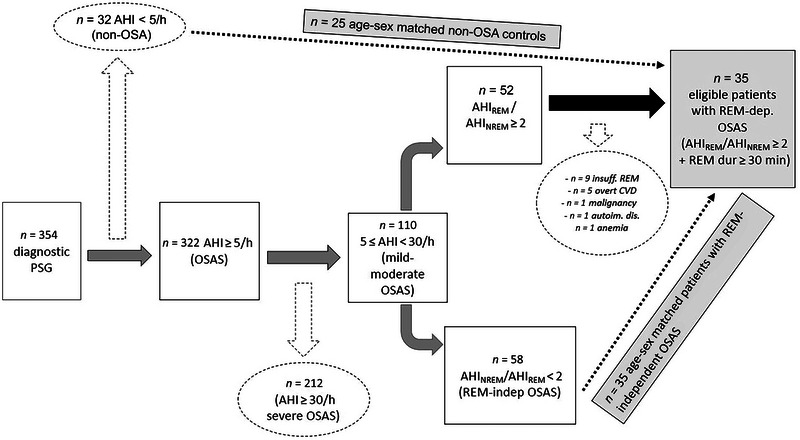
Flow chart regarding the sample selection. AHI, apnea–hypopnea index; autoim. dis., autoimmune disease; insuff. REM, insufficient REM duration; OSA, obstructive sleep apnea; OSAS, obstructive sleep apnea syndrome; overt CVD, overt cardio‐cerebrovascular disease; REM, rapid eye movement.

In our sleep disorders unit, we evaluated 354 patients with symptoms indicating OSAS between June 2017 and February 2019. Out of these patients, 322 were diagnosed with OSAS based on their full‐night diagnostic polysomnography (AHI ≥ 5/h), and 110 had a mild‐to‐moderate disease level (5 ≤ AHI < 30). Out of these 110 patients, 52 had a ratio of AHI_REM_/AHI_NREM_ greater than or equal to 2. After applying the exclusion criteria, we found that 35 patients were eligible for the primary definition of REM‐dependent OSAS. To test the main hypothesis, we consecutively selected 35 age–sex‐matched patients with mild‐to‐moderate REM‐independent OSAS and 25 non‐OSA controls (Figure 1).

The study population consisted of 95 patients with the following baseline demographic features: 38 (40%) were female, and 57 (60%) were male. The mean age was 50.62 ± 11.7 years, and the mean BMI was 30.06 ± 5.03 kg/m2. Among them, 51 (54%) reported a history of smoking, while 37 (40%) had at least one of the following cardiovascular risk factors: hypertension (26%), diabetes mellitus (19%), and hyperlipidemia (18%). None of the patients had been diagnosed with overt cardio‐cerebrovascular disease such as acute myocardial infarction (MI), arrhythmias, atrial fibrillation (AF), congestive heart failure (HF), or stroke, as mentioned in the methods section. The median ESS score of the entire study population was 4 (ranging from 0 to 20).

### 3.1 Analysis of patients with REM‐dependent OSAS, REM‐independent OSAS, and non‐OSA controls according to primary definition

There was no difference in terms of age, gender, BMI, smoking history, prevalence of cardiovascular risk factors, and ESS scores among three groups (p = .683, .218, .954, .937, .343, and .584, respectively). The demographic and laboratory parameters of the study population are presented in Table 1.

**TABLE 1:** Baseline characteristics and examination results of the study population.

	REM‐independent OSAS (*n* = 35)	REM‐dependent OSAS_(primary)_ (*n* = 35)	Non‐OSAS controls (*n* = 25)	*p* value
Age (years)	51.2 ± 12.2	49.6 ± 12.7	48.5 ± 11.8	.683
Gender, male (%)	71% (*n* = 25)	54% (*n* = 19)	52% (*n* = 13)	.218
BMI (kg/m^2^)	30.3 ± 5.3	30 ± 5.6	29.9 ± 3.7	.954
ESS	5 (0–12)	4 (0–20)	7.5 (1–17)	.584
Smoking history (%)	51% (*n* = 18)	54% (*n* = 19)	56% (*n* = 14)	.937
CVR prevalence (%)	49% (*n* = 17)	31% (*n* = 11)	40% (*n* = 10)	.343
Polysomnography—Sleep parameters				
TRT (min)	463.4 ± 38.9	455.3 ± 30.4	453.7 ± 32.5	.494
TST (min)	371.5 ± 49.5	366.8 ± 49.4	349.8 ± 62.9	.449
Sleep efficiency (%)	80.4 ± 8.8	80.8 ± 8.7	76.9 ± 12.1	.284
SOL (min)	14.6 (3.7–128.4)	13.5 (6.6–60.4)	18.4 (5.2–55.3)	.155
Arousal index (%)	**23.7 (7–71.9)**	15.4 (0.9–62.7)	10.9 (3.1–30.2)	<.001*
WASO (min)	62.2 (15–181.1)	60.5 (18.5–173)	65.8 (3.5–189.1)	.869
N1 stage (%)	**29.5 ± 10.6**	19.9 ± 8.3	17.1 ± 8.4	<.001*
N2 stage (%)	42.9 ± 9.5	44.8 ± 10.4	48.9 ± 9.9	.073
N3 stage (%)	15.5 ± 8.3	19.1 ± 9.2	19.1 ± 9.5	.171
REM stage (%)	**12.1 ± 6.1**	**16.2 ± 5.4**	13.2 ± 6.8	.015*
REM duration (min)	42.7 (3.4–128.4)	52.9 (30.3–122.1)	42.1 (4.7–135.7)	.033*
Polysomnography—Respiratory parameters				
OSA level (mild, %)	26% (*n* = 9)	40% (*n* = 14)	**–**	.203
AHI_REM_ (/h)	**18.5 (0–49.8)**	**43 (9.4–70.1)**	**4.5 (0–27.7)**	<.001*
AHI_NREM_ (/h)	**18 (5.3–50.7)**	**12.4 (3–25.3)**	**2.3 (0.4–4.9)**	<.001*
AHI_Total_ (/h)	18.6 ± 6.6	16.1 ± 6.9	**2.8 ± 1.4**	<.001*
ODI	21.6 (3.6–41.4)	15.6 (0.8–39.1)	**3.6 (0–4.3)**	<.001*
spO_2_ < 90% (%)	1.6 (0–41.7)	2.1 (0–41.1)	**0 (0–17.3)**	.001*
Minimum spO_2_	84 (66–93)	83.5 (70–93)	**89 (73–96)**	.001*
Average spO_2_	93.2 ± 1.6	93 ± 1.6	**94.3 ± 1.8**	.008*
Inflammatory indices				
PDW	13.2 ± 1.8	**13.5 ± 2**	**12.2 ± 1.9**	.025*
SII	543.3 ± 220.3	**587.2 ± 239.6**	**441.8 ± 133.2**	.032*

*Note*: *p* value belongs to analysis of variance (except for OSA level); groups showing significant differences are highlighted in bold and results of post hoc tests and/or pairwise comparisons are detailed in the text.

Abbreviations: AHI, apnea–hypopnea index; BMI, body‐mass index; CVR, cardiovascular risk factor; ESS, Eppworth sleepiness scale; ODI, oxygen desaturation index; OSAS, obstructive sleep apnea syndrome; PDW, platelet distribution width; REM, rapid eye movement; REM‐dependent OSAS_(primary)_, REM‐dependent OSAS according to primary definition; SII, systemic immune‐inflammation index; SOL, sleep onset latency; spO_2_ < 90%, desaturation duration; TRT, total recording time; TST, total sleep time; WASO, wake after sleep onset.

*A two‐tailed *p* value of <.05 is considered statistically significant.

Regarding the sleep parameters of polysomnography, the percentage of N1 stage and arousal index (AI) were increased in patients with REM‐independent OSAS in comparison to the patients with REM‐dependent OSAS_(primary)_ and non‐OSAS controls (29.5 ± 10.6, 19.9 ± 8.3, 17.1 ± 8.4, p < .001 for N1 stage; 23.7 [7–71.9], 15.4 [0.9–62.7], 10.9 [3.1–30.2], p < .001 for AI, respectively). The proportion of REM sleep (%) and duration of REM sleep (minutes) in patients with REM‐dependent OSAS_(primary)_ was higher than that of REM‐independent OSAS (16.2 ± 5.4 vs. 12.1 ± 6.1, p = .015 for REM sleep proportion and 52.9 [30.3–122.1] vs. 42.7 [3.4–128.4], p = .022 for REM sleep duration, respectively). Other sleep measures, including TRT, TST, SE, SOL, WASO, and the ratio of N2 and N3 stages, were similar between the groups.

Regarding the respiratory measures, AHI_total_, AHI_REM_, and AHI_NREM_ were significantly higher in patients with OSAS in comparison to non‐OSA controls, irrespective of sleep‐stage dependency (p < .001). Although AHI_total_ was similar between patients with REM‐dependent and REM‐independent OSAS (p = .388), AHI_REM_ and AHI_NREM_ showed a significant difference between the two groups (43 [9.4–70.1] vs. 18.5 [0–49.8], p < .001 for AHI_REM_ and 12.4 [3–25.3] vs. 18 [5.3–50.7], p = .011 for AHI_NREM_, respectively). Furthermore, oximeter indices including ODI, desaturation duration, minimum O2, and average O2 were similar between the two groups with OSAS, but their comparison with non‐OSA controls displayed a difference in favor of the controls (p < .05).

Concerning the inflammatory indices, variance analyses yielded significance for both parameters including PDW and SII. (p = .025 and .032, respectively). Post‐hoc tests revealed an increase in PDW and SII among REM‐dependent OSAS_(primary)_ patients as compared to non‐OSA controls (p = .022 for PDW and p = .029 for SII, respectively). However, comparisons between two groups of OSAS and comparisons between patients with REM‐independent OSAS versus non‐OSA controls failed to demonstrate such significance (p = 1.00 and .161 for PDW, and p = 1.00 and .192 for SII, respectively).

After adjusting for BMI and cardiovascular risk, REM‐dependency still demonstrated an independent association with PDW and SII (Table 2).

**TABLE 2:** Coefficients of stepwise multiple linear regression analysis for platelet distribution width (PDW) and systemic immune‐inflammation index (SII).

PDW												
Variables	Model 1				Model 2				Model 3			
	*B*	*SE*	*β*	*t*	*B*	*SE*	*β*	*t*	*B*	*SE*	*β*	*t*
**BMI**	0.010	0.057	0.023	0.169	0.005	0.056	0.012	0.089	0.003	0.054	0.008	0.064
**CVR**					0.739	0.558	0.179	1.323	0.838	0.535	0.203	1.565
**REM dep**.									1.344	0.546	0.319	2.461*
**Adjusted R2**	‐0.018				‐0.004				0.083			
**F Change**	0.029				1.750				6.057*			
**F**	0.029				0.889				2.668*			

SII												
Variables	Model 1				Model 2				Model 3			
	*B*	*SE*	*β*	*t*	*B*	*SE*	*β*	*t*	*B*	*SE*	*β*	*t*
**BMI**	11.006	5.804	0.254	1.896	10.485	5.926	0.242	1.769	9.825	5.652	0.227	1.738
**CVR**					‐34.873	65.638	‐0.073	‐0.531	‐55.216	63.071	‐0.115	‐0.875
**REM dep**.									143.499	57.714	0.323	2.486*
**Adjusted R2**	0.047				0.033				0.122			
**F Change**	3.596				0.282				6.182*			
**F**	3.596				1.914				3.467*			

Abbreviations: PDW: Platelet distribution width, SII: Systemic immune‐inflammation index, BMI: Body‐mass index, CVR: Cardiovascular risk (referred to the presence of at least one the following: hypertension, diabetes mellitus, hyperlipidemia and smoking history), REM dep.:REM dependency (referred to the presence of AHI_REM_/AHI_NREM_≥2).

*Correlation is significant at the 0.05 level (two tailed).

Correlation analysis between PDW—AHI, PDW—ODI, SII—AHI, and SII—ODI did not demonstrate significance in patients with OSAS (PDW—AHI: r = −0.219, p = .068; PDW—ODI: r = −0.158, p = .191; SII—AHI: r = −0.003, p = .979; SII—ODI: r = −0.050, p = .685).

### 3.2 Analysis of patients with REM‐dependent OSAS according to different definitions

For the second purpose of the study, the study population was reclassified based on two additional definitions of REM‐dependent OSAS (Table 3), which were outlined in a relevant research by Conwell et al. (2012).

**TABLE 3:** Patient characteristics according to different definitions of rapid eye movement (REM)‐dependent obstructive sleep apnea syndrome (OSAS).

	Primary definition (*n* = 35)	Traditional definition (*n* = 23)	Strict definition (*n* = 11)	*p* value
Age (years)	49.6 ± 12.7	47.3 ± 13.4	50.6 ± 14.4	.734
Gender, male (%)	54% (*n* = 19)	56.5% (*n* = 13)	45.5% (*n* = 5)	.827
BMI (kg/m^2^)	29.9 ± 5.6	28.8 ± 6	27.8 ± 4.4	.371
ESS	4 (0–20)	4 (0–13)	4 (0–13)	.866
Smoking history(%)	54% (*n* = 19)	61% (*n* = 14)	64% (*n* = 7)	.811
CVR prevalance (%)	69% (*n* = 24)	78% (*n* = 18)	82% (*n* = 9)	.577
Sleep efficiency (%)	80.8 ± 8.7	82.1 ± 7.8	83.1 ± 8.8	.705
REM duration (min)	52.9 (30.3–122.1)	56.9 (30.4–116.1)	65.6 (33.1–116.1)	.746
Slow wave sl. (min)	68.1 (8.4–197.9)	75.4 (8.4–197.9)	65.5 (23–116)	.889
Arousal index (%)	15.4 (0.9–62.7)	14.8 (0.9–44.8)	14.1 (0.9–44.8)	.854
WASO (min)	60.5 (18.5–173)	59.5 (18.5–159.7)	33.5 (18.5–115)	.313
OSA level (mild, %)	**40% (*n*** = **14)**	56.5% (*n* = 13)	**91% (*n*** = **10)**	.012*
AHI_Total_ (/h)	**16.4 ± 7.1**	12.7 ± 4.5	**9.3 ± 3.6**	.002*
AHI_REM_ (/h)	41.7 ± 15.8	35.5 ± 14.8	30.6 ± 15.6	.079
AHI_NREM_ (/h)	**12.2 ± 6.3**	8.5 ± 3.8	5 ± 1.1	<.001*
PDW	13.5 ± 2	13.5 ± 1.9	13.7 ± 2	.980
SII	587.2 ± 239.7	598.9 ± 269.6	649.7 ± 282.6	.782

*Note*: *p* value belongs to analysis of variance (results of post hoc tests and/or pairwise comparisons are detailed in the text), groups showing significant differences are highlighted in bold.

Abbreviations: AHI, apnea–hypopnea index; BMI, body‐mass index; CVR prev., cardiovascular risk factor prevalence; ESS, Eppworth sleepiness scale; PDW, platelet distribution width; SII, systemic immune‐inflammation index; Slow wave sl., slow wave sleep (N3); WASO, wake after sleep onset.

*A two‐tailed *p* value of <.05 is considered statistically significant.

As mentioned above, 35 patients (37%) in the current study cohort were already diagnosed with REM‐dependent OSAS_(primary)_ according to primary criteria. This rate decreased to 24% (n = 23) while using traditional criteria (overall AHI ≥ 5, AHI_REM_/AHI_NREM_ ≥ 2, and AHI_NREM_ < 15) and further decreased to 12% (n = 11) after application of strict criteria (overall AHI ≥ 5, AHI_REM_/AHI_NREM_ ≥ 2, and AHI_NREM_ < 8). When compared to the patients satisfying strict criteria, AHI_total_ significantly increased, and proportion of mild disease was accordingly decreased in patients meeting primary criteria (p = .002 and .012, respectively). Similarly, AHI_NREM_ was higher for the patients included in the primary definition group (p < .001) as compared to the other two groups (p = .026 for traditional and p < .001 for strict definition, respectively). While there was a tendency for AHI_REM_ to increase from the strict definition to the primary one, this difference was not statistically significant (p = .079). Other features, such as baseline demographics, clinical characteristics, remaining sleep parameters, and inflammatory indices, were similar among the three groups when considering different definitions of REM‐dependent OSAS (p > .05).

### 3.3 Analysis between patients with REM‐dependent OSA versus REM‐independent OSA and non‐OSA controls according to traditional and strict definitions

#### 3.3.1 Traditional definition

As patients were classified based on the traditional definition of REM‐dependent OSAS, 23 (24%) were diagnosed with REM‐dependent OSAS(traditional) while 47 (50%) were diagnosed with REM‐independent OSAS. The control group consisted of 25 individuals and was not affected by the definition of REM‐dependent OSAS according to the study's design. Patients with REM‐independent OSAS were found to have a higher proportion of N1 sleep stage and increased AI when compared to patients with REM‐dependent OSAS_(traditional)_ and non‐OSA controls. Specifically, the percentage of N1 stage was 27.2 ± 10.8 for patients with REM‐independent OSA, which was significantly higher than 19.5 ± 8.2 for patients with REM‐dependent OSA_(traditional)_ (p = .007) and 17.5 ± 8.5 for non‐OSA controls (p < .001). Similarly, the AI for patients with REM‐independent OSA was 23.4 (3–71.9), which was significantly higher than 14.8 (0.9–44.8) for patients with REM‐dependent OSA (p = .01) and 10.7 (3.1–50.2) for non‐OSA controls (p = .001).

The AHI_Total_ varied significantly among three groups, with the REM‐independent OSAS group showing the highest mean (19.8 ± 6.6), followed by the REM‐dependent OSAS_(traditional)_ group (12.7 ± 4.5), while the lowest mean was observed in the control group (2.8 ± 1.4) (p < .001). The REM‐dependent OSAS_(traditional)_ group had a larger number of patients with mild disease when compared to the REM‐independent OSAS group (56.5% vs. 21%, p = .003).

In patients with REM‐dependent OSAS_(traditional)_, both PDW and SII were found to be elevated as compared to non‐OSA controls. The values for PDW were 13.5 ± 1.9 and 12.9 ± 1.9 for REM‐dependent OSAS_(traditional)_ and non‐OSA controls, respectively, with a p‐value of .036. Similarly, the values for SII were found to be 598.9 ± 269.6 and 441.8 ± 133.2 for REM‐dependent OSAS_(traditional)_ and non‐OSA controls, respectively, with a p‐value of .034. However, no such significant difference was observed in patients with REM‐independent OSAS as compared to non‐OSA controls (Table 4).

**TABLE 4:** Analyses of patients with rapid eye movement (REM)‐dependent obstructive sleep apnea syndrome (OSAS), REM‐independent OSAS, and non‐OSAS controls according to traditional and strict criteria of “REM‐dependent OSAS.”

Traditional criteria regarding REM‐dependent OSAS				
	REM‐independent OSAS (*n* = 47)	REM‐dependent OSAS “traditional” (*n* = 23)	Non‐OSAS controls (*n* = 35)	*p* value
Age (years)	51.9 ± 11.9	47.3 ± 13.4	48.5 ± 11.8	.262
Gender, male (%)	66% (*n* = 31)	56% (*n* = 13)	52% (*n* = 13)	.478
BMI (kg/m^2^)	30.7 ± 5	28.8 ± 6	29.9 ± 3.7	.321
ESS	5 (0–20)	4 (0–13)	7.5 (1–17)	.428
Smoking hist.(%)	58% (*n* = 33)	61% (*n* = 14)	56% (*n* = 14)	.942
CVR prev. (%)	49% (*n* = 23)	22% (*n* = 5)	40% (*n* = 10)	.093
N1 stage (%)	**27.2 ± 10.8**	19.5 ± 8.2	17.5 ± 8.5	<.001*
N3 stage (%)	16 ± 8.4	20 ± 9.5	18.9 ± 8.3	.151
Arousal Index (%)	**23.4 (3–71.9)**	14.8 (0.9–44.8)	10.7 (3.1–50.2)	<.001*
OSA level (mild, %)	**21% (*n*** = **10)**	**56.5% (*n*** = **13)**	–	.003*
AHI	**19.8 ± 6.6**	**12.7 ± 4.5**	**2.8 ± 1.4**	<.001*
PDW	13.2 ± 1.9	**13.6 ± 1.9**	**12.2 ± 1.9**	.028*
SII	549.1 ± 208.9	**598.9 ± 269.6**	**441.8 ± 133.2**	.031*

**Strict criteria regarding REM‐dependent OSAS**				
	REM‐independent OSAS (*n* = 59)	REM‐dependent OSAS “strict” (*n* = 11)	Non‐OSA controls (*n* = 35)	*p* value
Age (years)	50.4 ± 12.1	50.6 ± 14.4	48.5 ± 11.8	.795
Gender, male (%)	66% (*n* = 39)	45% (*n* = 5)	52% (*n* = 13)	.279
BMI (kg/m^2^)	30.5 ± 5.5	27.8 ± 4.4	29.9 ± 3.7	.254
ESS	5 (0–20)	4 (1–13)	7.5 (1–17)	.536
Smoking history(%)	51% (*n* = 30)	64% (*n* = 7)	56% (*n* = 14)	.711
CVR prev. (%)	44% (*n* = 26)	18% (*n* = 2)	40% (*n* = 10)	.274
N1 stage (%)	**25.7 ± 10.7**	19.3 ± 8.6	**17.5 ± 8.5**	.001*
N3 stage (%)	17.1 ± 9.2	18.2 ± 7.4	18.9 ± 8.3	.643
Arousal index (%)	**23.1 (3–71.9)**	14.1 (0.9–44.8)	**10.7 (3.1–50.2)**	.003*
OSA level (mild, %)	**22% (*n*** = **13)**	**91% (*n*** = **10)**	–	.003*
AHI	19.03 ± 6.3	9.3 ± 3.6	2.8 ± 1.4	<.001
PDW	13.3 ± 1.9	**13.7 ± 2**	**12.2 ± 1.9**	.030*
SII	549 ± 217	**649.7 ± 282.6**	**441.8 ± 133.2**	.016*

*Note*: *p* value belongs to analysis of variance (except for OSA level); groups showing significant differences are highlighted in bold and results of post‐hoc tests and/or pairwise comparisons are detailed in the text.

Abbreviations: AHI, apnea–hypopnea index; BMI, body‐mass index; CVR prev., cardiovascular risk factor prevalence; ESS, Eppworth sleepiness scale; PDW, platelet distribution width; SII, systemic immune‐inflammation index.

*A two‐tailed *p* value of <.05 is considered statistically significant.

#### 3.3.2 Strict definition

Using the strict criteria, 11 individuals (12%) were diagnosed with REM‐dependent OSAS_(strict)_, while 59 were diagnosed with REM‐independent OSAS and 25 were non‐OSA controls. Patients with REM‐independent OSA had the highest AHI_Total_ score (19.03 ± 6.3), followed by REM‐dependent OSA_(strict)_ (9.3 ± 3.6) and non‐OSA controls (2.8 ± 1.4) (p < .001). The rate of patients with mild disease was significantly higher in the REM‐dependent OSAS_(strict)_ group compared to the REM‐independent OSAS group (91% vs. 22%, p < .001).

REM‐independent OSAS patients had higher N1 stage and AI compared to non‐OSA controls (25.7 ± 10.7 vs. 17.5 ± 8.5, p = .002 and 23.1 [3–71.9] vs. 10.7 [3.1–50.2], p = .006 for AI). The difference between patients with REM‐dependent OSAS_(strict)_ and REM‐independent OSAS was not significant.

PDW and SII were higher in REM‐dependent OSAS(strict) patients compared to non‐OSA controls (13.7 ± 2 vs. 12.2 ± 1.9, p = .039 for PDW and 649.7 ± 282.6 vs. 441.8 ± 133.2, p = .020 for SII, respectively). This relationship was not observed between REM‐independent OSAS and non‐OSA controls (Table 4).

## 4 DISCUSSION

The current findings demonstrate that PDW and SII are increased in mild‐to‐moderate REM‐dependent OSAS as compared to non‐OSA controls, regardless of the definition used. Such an elevation was not true for patients with REM‐independent OSAS and was sustainably significant after adjustment for potential confounders.

Both PDW and SII have been proven to be diagnostically and prognostically useful in acute and chronic conditions where inflammation plays a critical role. Therefore, it is reasonable to suggest that REM‐dependent OSAS reflects a detrimental status even in mild‐to‐moderate disease levels, in terms of underlying inflammation, as opposed to non‐OSAS individuals (Adam et al., 2015; Gur et al., 2022; Nena et al., 2012; Yi et al., 2021).

REM‐dependent OSAS is a separate subtype of OSAS, which constitutes 12%–36% of all cases and has some typical features such as preference for female gender, younger age, and milder disease level (Haba‐Rubio et al., 2005; Mano et al., 2019; Mokhlesi, 2012; Nieto et al., 2000). The physiologic characteristics of the REM stage itself include diminished muscle tone, weakened genioglossus reflex activity in response to negative pressure, and impaired chemosensitivity that provides a natural basis for easier collapsibility of the upper airway (Eckert & Malhotra, 2008). This results in longer respiratory events together with profound hypoxemia, which sounds dreadful at the beginning. However, arguments on cyclic intermittent hypoxia which is claimed to have a stronger impact on inflammatory pathways rather than the sustained pattern characteristic for REM‐dependent OSAS, as well as the increased proportion of patients with milder disease in this phenotype, cause a dilemma whether REM‐dependent OSAS is a relatively benign phenomenon or a deleterious variant (Garvey et al., 2009; Mokhlesi, 2012; Ryan et al., 2005). Furthermore, this insistent hypoxia was suggested to facilitate the development of an adaptive response that serves the purpose of improvement in tissue perfusion and oxygenation, according to some reports (Garvey et al., 2009; Ryan et al., 2005). Nevertheless, our results raised the need to revisit this underrated variant and its undesirable consequences one more time.

A number of studies have linked REM‐dependent OSAS with cardiovascular morbidity. For example, Mokhlesi et al. (2014, 2015) found a significant correlation between REM‐AHI and prevalent hypertension, as well as a non‐dipping pattern, in longitudinal analysis of the Wisconsin sleep cohort. Independent associations between severe REM OSAS and hypertension were also discovered by Appleton SL et al. in the MAILES cohort (Appleton et al., 2016). Another study of 518 patients with mild OSAS found a higher prevalence and risk of cardiometabolic diseases, especially hypertension, among those with REM‐OSAS (Sangchan et al., 2023). Koo et al. (2020) suggested the predictive potential of moderate‐severe OSA during REM sleep for the presence of metabolic syndrome, whereas Grimaldi et al. (2014) showed an independent association between the increment of REM‐AHI and HbA1c. Recently, Ljungren et al. soon demonstrated increased values of carotid intima thickness as a sign of atherosclerosis among severe REM sleep apnea sufferers, females in particular (Ljunggren et al., 2022).

On the other hand, studies examining such link in terms of inflammation perspective are very few. In a retrospective analysis of 100 patients with allergic rhinitis, an immunoglobulin E‐related inflammatory condition, subjects with a moderate to severe REM‐RDI were reported to have 5.1 times more likelihood of being allergic when compared to the ones with a normal to mild range of a REM‐RDI (Berson et al., 2018). The assessment of inflammatory markers in 33 females in the 3rd trimester, of whom 11 had a diagnosis of OSA, indicated significant correlations between AHIREM and TNF‐α, IL‐1ß, IL‐6, and IL‐8 (Alonso‐Fernandez et al., 2021). Somewhat similar results were reported by Gurpinar et al. (2020) through a different marker, where the authors stated increase in the neutrophil‐to‐lymphocyte ratio (NLR) among patients with REM‐related OSAS when compared to those with Supine‐related OSAS and habitual snorers.

To our knowledge, our study is the first to investigate SII and PDW in REM‐dependent OSAS and point out significant elevation in this phenotype.

Hypoxia during respiratory events is considered to be the leading mediator underlying systemic inflammation in OSAS, and it is known to increase the turnover of platelets resulting in subsequent alterations in morphology (Korniluk et al., 2019). Sympathetic overactivity, another prerequisite, contributes to the pathway of platelet activation additionally (Kurt & Yildiz, 2013). Platelet distribution width (PDW) is considered to be a steady indicator of this process (Vagdatli et al., 2010). “Systemic immune‐inflammation index” (SII) holds information from both platelets and white blood cell components, as the conductor of inflammation, and is suggested to provide further prescience in terms of vascular consequences (Gur et al., 2022; Yi et al., 2021). For this reason, these two markers were given priority during this study. Although this activation process could be represented through specific markers like soluble CD40 ligand and soluble P‐selectin, the markers we assessed were simple and easily available, and that was another reason to be preferred (Akinnusi et al., 2009; Shimizu et al., 2002; Wu et al., 2018).

Regarding the additional findings of the current study, our results showed a gradual decrease in AHI_TOTAL_ and AHI_NREM_, accompanied by an increase in the proportion of mild disease as the criteria for REM‐dependent OSAS became more stringent. This outcome was expected because stricter criteria require a lower limit of AHI during NREM sleep, which constitutes a significant portion of the night. These findings are consistent with previous reports on the subject (Al Oweidat et al., 2018; Arslan et al., 2020; Conwell et al., 2012; Hoshino et al., 2020). Patients with REM‐independent OSAS in this study were found to have increased AI and spent more time in N1 stage when compared to the ones with REM‐dependent OSAS according to primary and traditional definitions. It could be explained by the higher number of respiratory events elicited in patients with REM‐independent OSAS, as reflected by AHI, although the difference did not reach significance within the scope of the primary definition. It is reasonable for a sleep, which is exposed to more respiratory events, to be more fragmented and superficial.

Patients with REM‐dependent OSAS have been reported to experience lower arousal thresholds and insomnia instead of excessive daytime sleepiness. In 2020, Hoshino et al. (2020) conducted a study on a large group of participants and found a significant association between REM‐related OSA and increased Pittsburgh Sleep Quality Index (PSQI) scores in all adjusted models. The same group also discovered that patients with REM‐related OSA have a lower arousal threshold, which prevents them from reaching the deeper sleep stage where stable breathing is observed (Hoshino et al., 2022). The two conditions that are reported frequently in this phenotype could amplify the aforementioned process through oxidative stress. A rise in malondialdehyde, as an indicator of oxidative stress, and a diminishment in glutathione‐peroxidase activity, as one of the most important scavengers of free oxygen radicals, were demonstrated in individuals with primary insomnia previously (Gulec et al., 2012). In another study that investigated the effect of modification of hypoxic exposure on oxidative stress, loop gain, and the arousal threshold in 13 males with OSAS, a positive correlation between levels of 8‐hydroxy‐2‐deoxy guanosine (8‐OHdG‐OHdG), a well‐established biomarker of oxidative stress, and number of arousals were already stated (Panza et al., 2019). Taken together, low arousal threshold and insomnia, which were not specifically examined during this study, also appear to contribute to the underlying systemic inflammation in REM‐dependent OSAS. 

There are some limitations to the current study. First, the sample size was relatively small and could not be augmented due to the retrospective design. Secondly, some other aspects of REM‐dependent OSAS, like frequent associations with mood disorders, were not given priority and not discussed in detail, as this was not the main focus, although they were questioned in the context of comorbid diseases, medication history, and habits in the first visit (BaHammam et al., 2023). Finally, patients were assigned to the REM‐dependent OSAS group based on widely used definitions in the literature, as there was still no universally accepted definition for the entity at the time of this study (Conwell et al., 2012; Hoshino et al., 2020).

To sum up, mild‐to‐moderate REM‐dependent OSAS is associated with a significant inflammatory predisposition, which is not as noticeable for the stage‐independent form (Ryan et al., 2005; Wu et al., 2018). This relationship is independent of confounding factors and remains consistent even after using different definitions. Based on these findings, patients with REM‐dependent OSAS should be carefully evaluated and monitored for the consequences of this increased inflammatory status. Prospective studies with larger sample sizes would be beneficial in further clarifying the clinical implications of this association.

## AUTHOR CONTRIBUTIONS

**Asli Akyol Gurses**: Conceptualization; data curation; formal analysis; investigation; methodology; supervision; writing—original draft; writing—review and editing. **Utku Ogan Akyildiz**: Conceptualization; investigation; methodology; supervision; writing—review and editing.

## CONFLICT OF INTEREST STATEMENT

The authors declare no conflicts of interest.

### PEER REVIEW

The peer review history for this article is available at https://publons.com/publon/10.1002/brb3.3546

## Data Availability

Data regarding this study are available upon reasonably request from the corresponding author
